# TRP Channels in the Focus of Trigeminal Nociceptor Sensitization Contributing to Primary Headaches

**DOI:** 10.3390/ijms21010342

**Published:** 2020-01-04

**Authors:** Mária Dux, Judit Rosta, Karl Messlinger

**Affiliations:** 1 Department of Physiology, University of Szeged, Dóm tér 10, H-6720 Szeged, Hungary; tothne.rosta.judit.maria@med.u-szeged.hu; 2 Institute of Physiology and Pathophysiology, Friedrich-Alexander-University Erlangen-Nürnberg, Universitätsstr. 17, D-91054 Erlangen, Germany; karl.messlinger@fau.de

**Keywords:** transient receptor potential vanilloid 1, transient receptor potential ankyrin 1, calcitonin gene-related peptide, primary headaches, trigeminal system, nociception

## Abstract

Pain in trigeminal areas is driven by nociceptive trigeminal afferents. Transduction molecules, among them the nonspecific cation channels transient receptor potential vanilloid 1 (TRPV1) and ankyrin 1 (TRPA1), which are activated by endogenous and exogenous ligands, are expressed by a significant population of trigeminal nociceptors innervating meningeal tissues. Many of these nociceptors also contain vasoactive neuropeptides such as calcitonin gene-related peptide (CGRP) and substance P. Release of neuropeptides and other functional properties are frequently examined using the cell bodies of trigeminal neurons as models of their sensory endings. Pathophysiological conditions cause phosphorylation, increased expression and trafficking of transient receptor potential (TRP) channels, neuropeptides and other mediators, which accelerate activation of nociceptive pathways. Since nociceptor activation may be a significant pathophysiological mechanism involved in both peripheral and central sensitization of the trigeminal nociceptive pathway, its contribution to the pathophysiology of primary headaches is more than likely. Metabolic disorders and medication-induced painful states are frequently associated with TRP receptor activation and may increase the risk for primary headaches.

## 1. Anatomical Basis of Headache Generation

Primary headaches such as tension-type headache, migraine or trigemino-autonomic headaches are clinically well characterized, however, despite intense basic and clinical research, their origin remains still largely in the dark. In this contribution we ask whether sensitization of nociceptors may contribute to the generation of primary headaches [1] and how transduction channels of the transient receptor potential (TRP) type, which are typically expressed in nociceptors [2], are involved in the expected sensitizing mechanisms. Trigeminal nociceptors are primary afferents originating in the trigeminal ganglion, which is located in the Meckel’s cave, a cavern on the floor of the middle cranial fossa. The trigeminal ganglion houses the somata of the primary afferent neurons and gives rise to three large cranial nerves containing mainly the peripheral axons of pseudo-unipolar primary afferent neurons, the ophthalmic (V1), the maxillary (V2), and the mandibular (V3) nerve. Like all other nociceptors, the trigeminal nociceptors consist of small primary sensory neurons with unmyelinated C- and thinly myelinated Aδ-fibers, the central terminals of which run through the trigeminal nerve, enter the pontine brainstem and synapse on neurons in the spinal trigeminal nucleus (STN). The STN is also called trigemino-cervical brainstem complex, because it passes into the upper cervical segments without clear structural and functional boundary [3,4].

It is generally held that physiological pain results from activation of nociceptors innervating peripheral tissues, whereas pathophysiological pain may originate from more central sites of the nociceptive pathways. The question whether primary headaches belong to one of these categories is unsolved but most researchers may agree that trigeminal nociceptors are involved in the generation of headaches [1,5]. It is also widely accepted that nociceptors involved in headache generation innervate the meninges, although a contribution of nociceptors innervating tissues outside the head and the neck seems likely (see below). The cranial meninges consist of a delicate inner layer, the pia mater covering the entire cortical surface, and a tough outer layer bordering the skull, and the dura mater, which forms also the wall of the cranial sinuses. Between dura and pia mater, the cobweb-like arachnoidea lines the subarachnoidal space filled with cerebrospinal fluid. All three trigeminal partitions (V1–V3) participate in innervating the meninges, in the occipital region complemented by cervical afferents from segments C1–C3. In addition, the meninges are densely innervated also by postganglionic sympathetic fibers and sparsely by parasympathetic fibers originating in the cranial ganglia [4].

## 2. Morphology and Role of Meningeal Nociceptors in Headache Generation

The well-known classical intraoperative experiments performed by the groups of Harold Wolff and Wilder Penfield influenced our understanding of headache generation significantly until recently [6,7]. They observed that headache-like sensations could be elicited from arterial vessels of the dura mater, dural sinuses and large cerebral arteries, whereas the pia mater was entirely painless, regardless which noxious stimuli (mechanical, thermal or chemical) were used to stimulate these structures. There was a discrepancy with morphological data for a long time, since not only dural but also pial arteries were found to be innervated by thin peptidergic nerve fibers [8,9], which are regarded as nociceptive (see below). This discrepancy has been solved in recent years, since a French group of neurosurgeons has collected conclusive data showing that stimulation of pial arterial structures can well cause painful sensations [10]. In addition to intracranial structures, Wolff’s group reported long ago that also some extracranial arteries like the temporal artery can be sources for headache sensations [11], which is interesting in the light of new morphological findings of a collateral innervation of intra- and extracranial afferents [12,13].

**Figure 1:**
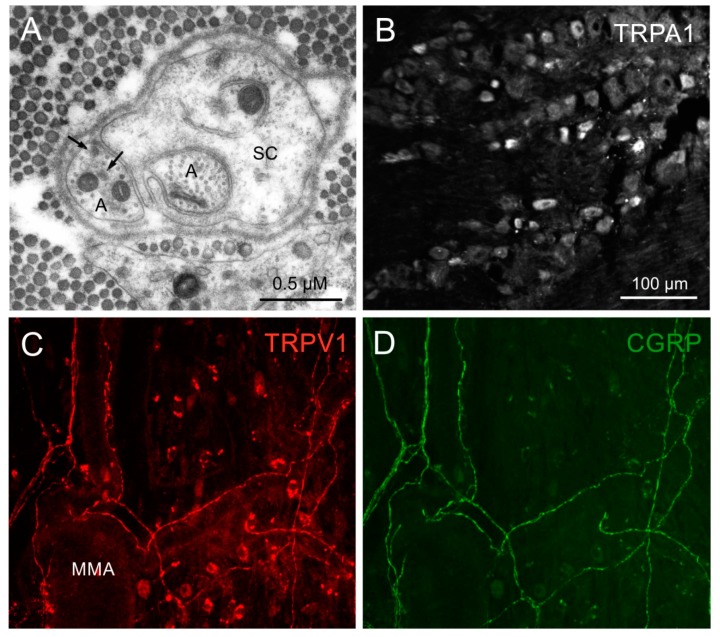
Ultrastructural characteristics of meningeal nociceptors. (**A**) Electron micrograph of a cross-sectioned small peripheral nerve fiber bundle in rat dura mater. Two sensory axons (A) are fully or partly wrapped by a Schwann cell (SC). The left axon contains two mitochondria and some dense-core vesicles (arrows), which most likely contain neuropeptides. The not covered axon membrane exposed to the surrounding (consisting mainly of collagen fibers) is probably an area of sensory transduction and neuropeptide release (see Figure 2). (**B**) Immunohistochemical staining of rat trigeminal ganglion for TRPA1. TRPA1-immunoreactive neurons are preferably small or middle-sized. (**C**–**D**) Double immunohistochemical staining for TRPV1 and calcitonin gene-related peptide (CGRP) in rat dura mater. Regarding the network of thin afferent fibers accompanying the middle meningeal artery (MMA) and its branches, TRPV1 and CGRP immunofluorescence are nearly completely co-localized.

How are meningeal C- and Aδ-nociceptors characterized morphologically? Like other nociceptors they form so-called free nerve endings, which in ultrastructural dimensions are sensory axons, frequently in form of small Remak bundles, i.e., several axons embedded in a more or less continuous sheath of Schwann cells [8,14]. In the course of the sensory endings innervating peripheral tissues the Schwann cells show gaps, free areas, where the axolemma has direct contact to the surrounding tissue, which justifies the term “free” nerve ending (Figure 1A). It is obvious that these free areas are the sites for neuropeptide release into the surrounding and the sites where the axon exhibits transduction molecules and is sensitive to environmental noxious stimuli [8]. The accompanying Schwann cells seem to have also important functions, which may include signaling between axon and Schwann cell. Assuming that the size and shape of the free axonal areas is variable and can be regulated by the Schwann cell, it may be speculated that an increase in these gaps can increase the sensitivity of the nociceptive fiber. Recently it has been reported that specialized glial cells associated with unmyelinated nociceptive nerve fibers in the skin act even as primary transducers, since they show mechanosensitive properties and transmit the nociceptive information to the nerve endings [15]. The above morphological characteristics of free nerve endings have been reported in several tissues such as articular ligaments [16,17], skin [18], teeth [19] and meninges [14,20]; though it is not yet clear if they characterize specifically nociceptors, because thermoreceptive nerve fibers form also free nerve endings. Thus, it is possible that the morphology of free nerve endings does not provide any modality-specific characteristics, so that it is rather the equipment with membrane-bound receptor molecules that determines the modality (see below).

The question if, and in which way, the activity of meningeal nociceptors determines the intensity of headaches is a matter of speculation. Meningeal nociceptors are certainly highly active when the dura mater is lesioned or inflamed, like in meningitis, since inflammatory mediators are very effective in driving primary and secondary trigeminal activity [21,22]. On the other hand, primary headaches like migraine and cluster headache can be extremely intense but we have no information about the level of primary afferent activity in these states. Following experimental cortical spreading depression (CSD) in rodents, which is regarded as an animal model of the aura phase followed by migraine attacks, i.e., a “natural” model of migraine generation, the meningeal C-fiber activity is remarkably low [23]. Therefore, it seems to be rather a matter of central sensitization that decides upon the intensity of headaches (see below). It may also be that the convergent afferent input from different tissues to the second order neurons in the spinal trigeminal nucleus (trigemino-cervical complex) is most important for the character and the intensity of headaches [24]. For example, strong afferent input from pericranial muscles and neck muscles is possibly not generating headaches unless there is additional meningeal afferent input. In a rat model of meningeal nociception, local anesthesia of neck muscles dramatically reduced the activity of spinal trigeminal neurons with convergent afferent input from the meninges [25].

## 3. The Trigeminal Ganglion Neuron as a Model for Its Peripheral and Central Terminals

Due to their small diameters, peripheral and central terminals of C- and Aδ-fibers are not accessible for several essential methods in neuroscience. For example, immunofluorescent intracellular or membrane-bound molecules associated with C-fibers can be visualized with confocal imaging in transparent tissues such as the rodent dura mater (Figure 1C,D) but quantifying immunofluorescence is not reliable in these tissues. Extracellular single fiber recordings can be performed in meningeal tissues with some special techniques like the tracking method [26,27] but measuring membrane potentials or currents with micropipettes is virtually impossible. Therefore molecular and neurophysiological properties of meningeal afferents are frequently studied by imaging and recording from trigeminal ganglion neurons in situ, isolated ganglia or in cell culture [26,28,29]. In these cases the soma of the neuron is regarded as a model of its endings, which is justified in that intracellular and membrane-bound molecules produced in the cell body are delivered via axonal transport to the peripheral and central terminals, where transduction and neurotransmission take place. Nevertheless this concept should be taken with caution, because molecules can be modified or assembled to functional units only in the terminals (for example the calcitonin gene-related peptide, CGRP, receptor complex) and some molecules may be transported unilaterally towards the periphery or the central nervous system (for example brain-derived neurotrophic factor, BDNF). Based on this concept different experimental approaches can be applied:

### 3.1. Immunohistochemistry

The trigeminal ganglion has been examined with this technique to detect neuropeptides such as substance P and calcitonin gene-related peptide (CGRP), long before TRP channels have been cloned and characterized. Probably the earliest immunohistochemical stainings, shortly after a similar study about dorsal root ganglia had been published [30], showed 40% of rat trigeminal ganglion with CGRP- and 20% with substance P-immunoreactivity with nearly 100% co-localization [31]. In the human trigeminal ganglion nearly half of the neurons was found to be CGRP immunoreactive [32]. Significant proportions of trigeminal ganglion neurons show immunoreactivity for the transient receptor potential channels vanilloid 1 (TRPV1) and ankyrin 1 (TRPA1) (Figure 1B). In a comprehensive study on human trigeminal ganglia the number of neurons with co-localization of CGRP and TRPV1 was dependent on the age of the individuals with 61% in newborn and 35% in adults [33]. Expression of TRPV1 and neuropeptides seem to be closely related in pathological states, e.g., in inferior alveolar nerve lesion [34].

### 3.2. Neuropeptide Release

Trigeminal ganglia in situ or cultivated trigeminal ganglion cells from rodents are frequently used to measure the release of substances following stimulation with noxious agents that target TRPV1 and TRPA1 receptors [35,36]. Particularly quantification of CGRP release from trigeminal ganglia or trigeminal cell cultures induced by capsaicin is regarded as a parameter for trigeminal activation and sensitization [37,38]. CGRP release from trigeminal ganglia has also been used to quantify the effect of antinociceptive substances like non-steroidal anti-inflammatory drugs and to analyze the mode of their actions [39]. The release studies are confounded by the fact that the trigeminal ganglion is wrapped by a double layer of dura mater, which prevents the direct access of stimulating substances and may hinder the diffusion of released substances into the bath solution.

### 3.3. Electrophysiological Recordings

In intact animals the trigeminal ganglia can be accessed by recording electrodes pushed through the cerebral cortex. Using this method, reliable extracellular recordings can be made, while the location of receptive fields indicates the innervation territories of trigeminal neurons. Threshold lowering, increase in activity upon suprathreshold stimulation and expansion of receptive fields are indicating nociceptor sensitization [28].

### 3.4. Calcium Imaging

Finally, trigeminal ganglion cell cultures have frequently been used to study the molecular properties of neurons and responses to endogenous and environmental mediators using calcium imaging [36,37]. Agonists and antagonists specifically targeting TRP receptor channels are employed to find out the role of TRPs in the neuronal activation [40]. For example, the irritating effect of carvacrol and eugenol, ingredients of spices, were shown by calcium imaging to result from activation of TRPA1 [41]. Using DiI tracing, dental primary afferents have been shown responding to hyperosmotic stimuli with calcium transients, which were dependent on transient receptor potential melastatin 8 (TRPM8) activation [42].

## 4. Expression of TRPV1 and TRPA1 and Co-Expression with Neuropeptides

Receptor channels of the TRP family are expressed by significant proportions of trigeminal ganglion neurons [43,44]. TRP receptors form unspecific cation transduction channels in peripheral sensory endings and may also be involved in synaptic transmission at the central terminals [45].

Nociceptive afferents can be grouped into peptidergic neurons, most of which express vanilloid-sensitive transient receptor potential (TRPV1) channels, and non-peptidergic neurons, which are characterized by their isolectin B4 (IB4) binding. The first group is sensitive to nerve growth factor (NGF), while the second group is sensitive to glial cell-line derived neurotrophic factor (GDNF) during the development [46,47]. Apart from the expression of TRPV1, the two groups may be different in some minor physiological functions like neurotransmitter release [48]. However, this grouping seems not very clear-cut, since immunostaining has shown considerable overlap of TRPV1 and IB4 binding in primary afferent neurons of rat and mouse, particularly in the rat trigeminal ganglion [49]. Interestingly, the authors found 70% of CGRP-immunoreactive neurons colocalized with TRPV1 immunoreactivity (see Figure 1C,D), which was significantly more compared to dorsal root ganglia and may be one explanation for the dominant role of CGRP as a signaling neuropeptide in the trigeminal system.

**Figure 2:**
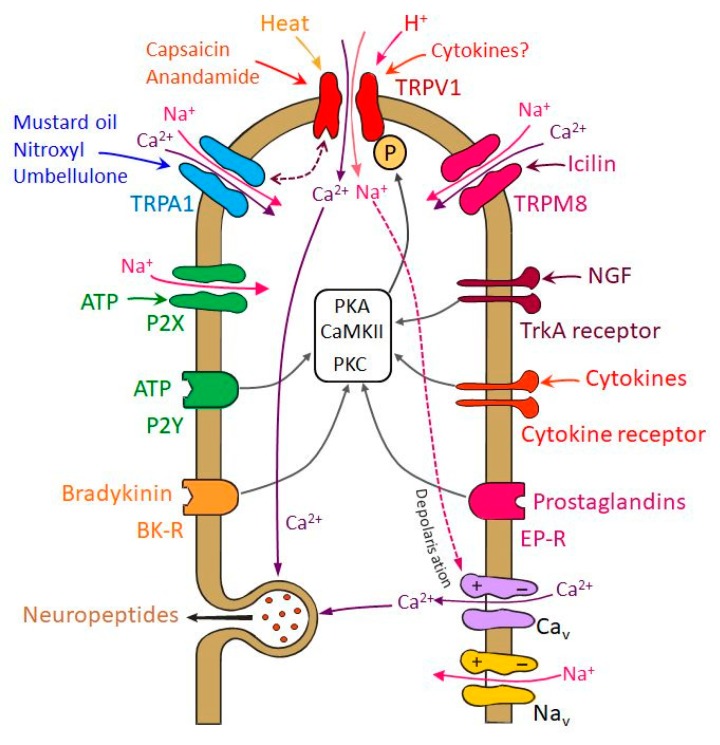
Scheme depicting important transduction processes in the nociceptive terminal. Transient receptor potential channels TRPV1, TRPA1 and TRPM8 may partly be co-expressed in the same terminals. TRPV1 is opened by noxious heat, whereas activation of TRPA1 and TRPM8 is facilitated under cold conditions. All TRP channels are also gated by specific endogenous and environmental substances. Opening of these transduction channels is followed by local cation inflow causing depolarization of the terminal, which can additionally activate voltage-gated cation channels inducing exocytosis of neuropeptides (Ca_v_) and generate action potentials (Na_v_). The TRP channels (here TRPV1 as an example) can be sensitized by phosphorylation (P) through several protein kinases (protein kinase A, PKA; protein kinase C, PKC; calcium calmodulin kinase II, CaMK II) induced by a variety of G-protein coupled receptors (purinergic receptors, P2Y; bradykinin receptors, BK-R; prostaglandin receptors, EP-R) or tyrosine kinase associated receptors (tyrosine kinase A, TrkA; cytokine receptors). TRP receptors can probably interact (double arrow between TRPV1 and TRPA1), thereby causing possibly both cross-sensitization and -desensitization.

TRPV1 receptor channels are activated by pungent substances like capsaicin or resiniferatoxin, noxious heat, acids (pH < 5.3), and different endogenous compounds including membrane-derived lipid metabolites like anandamide (Figure 2) [50]. TRPA1 receptor channels, which are highly colocalized with TRPV1 in trigeminal neurons, are activated by irritants like mustard oil and cannabinoids [51,52,53]. TRPA1 can also be activated by volatile substances such as umbellulone of the "headache tree" [54]. Its functional role in trigeminal nociception is controversial, because there is experimental evidence for a cooperative effect with TRPV1 in meningeal afferents [55] but also for a dual nociceptive-antinociceptive effect when recordings were made from spinal trigeminal neurons [56], as discussed below in more detail (Figure 2). Another member of the TRP receptor family implicated in the migraine pathophysiology is the TRPM8 that is a cold-sensitive channel activated also by natural and synthetic “cooling” agents, such as menthol and icilin (Figure 2). Some of the TRPM8-positive cells may also express TRPV1 or CGRP, but a substantial fraction is not labeled by nociceptive markers [57,58].

## 5. Functional Significance of Mediator Trafficking to Afferent Fiber Terminals

As discussed above, trigeminal ganglia or isolated ganglion neurons are frequently used as models of their peripheral (sometimes also central) terminals assuming that most of the signal molecules expressed in the cell bodies are delivered by axonal transport to the periphery and/or into the central nervous system. By this way neuropeptides are transported into the peripheral terminals, where they can be released upon stimulation by calcium-dependent exocytosis and induce symptoms of neurogenic inflammation [59,60]; likewise in the central terminals they act as neuromodulators contributing to synaptic transmission [61,62]. Receptors are transported as well and integrated into the terminal cell membrane, for example serotonin 1B/D (5-HT1B/D) receptors, the activation of which counteracts neuropeptide and glutamate release and hence eripheral transduction and central neurotransmission [63,64].

However, morphological and functional findings suggest that some of the receptor proteins are unidirectionally transported to a large extent. For example, CGRP receptor components have been found by confocal immunohistochemistry co-localized with axonal markers only in the central but not in the peripheral processes of rat trigeminal neurons, which may indicate that they are unilaterally delivered to the central terminals [65], although other data do not support this assumption [66]. As all these molecules are produced in the cell body, every mechanism interacting with their gene expression has consequences for the peripheral and central functions of these neurons. For example, CGRP receptors integrated into the presynaptic membrane of central trigeminal terminals may be activated by CGRP released from adjacent terminals of other trigeminal afferents to facilitate neurotransmitter release and synaptic transmission [67,68]. Another example is BDNF, the expression of which is enhanced by CGRP in cultured trigeminal neurons [69]. BDNF and other neurotrophic factors have been found to stimulate the expression of TRPV1 and to increase the sensitivity of TRPV1 and TRPA1 receptor channels in dorsal root ganglion neurons [70] as well as TRPV1-mediated CGRP release from trigeminal ganglion neurons [47]. BDNF is not specific for the somatosensory system but is also important for other, e.g., cardiovascular functions [71]. Interestingly, however, BDNF produced by trigeminal afferents is delivered by axonal transport into the trigeminal nucleus, where it can be released from central presynaptic terminals and may act on pre- and postsynaptic tyrosine kinase (TrkB) receptors to facilitate nociceptive transmission [72]. Thus, substances acting on neurons within the trigeminal ganglion, which is outside the blood-brain barrier, can have considerable impact on the peripheral and central functions of nociceptive transduction and transmission. This mode of action is clearly relevant regarding the recent discussion about big molecules like monoclonal antibodies, which reach the central nervous system only at very low concentrations [73] and are therefore assumed to act mainly outside the blood-brain barrier inhibiting CGRP signaling and reducing trigeminal functions involved in migraine [74], as reviewed elsewhere [75,76].

## 6. Signals Sensitizing Trigeminal Nociceptors—Involvement and Interaction of TRP Receptors

A lower threshold for neuronal activation, indicated as lowered pain threshold of facial areas one day prior to a migraine attack, can be observed in migraineurs compared with healthy controls suggesting an altered condition of neuronal excitability [77]. This may occur as result of peripheral and/or central sensitization of the trigeminal nociceptive pathway. During the initial phase of a migraine attack the occurrence of throbbing pain may be considered as the consequence of an increased sensitivity of peripheral trigeminal neurons that innervate the meninges [21]. Physical activities increasing intracranial pressure generally worsen these symptoms. Previously innocuous stimuli on the facial skin may be uncomfortable or even painful during a migraine attack. This cutaneous allodynia is thought developing through the sensitization of second-order neurons in the spinal trigeminal nucleus, which receive convergent input from meningeal tissues as well as from the facial skin [78,79]. Sensitization of primary sensory neurons may occur through repeated or sustained activation that may have also a long-lasting effect on the function of the second-order neurons in the trigeminal nociceptive pathway leading to an increased susceptibility even in the absence of the peripheral sensitizing agent.

Multiple changes in TRPV1 and TRPA1 receptor function may be involved in sensitization of trigeminal nociceptors (Figure 2). Phosphorylation of the receptors by protein kinase A (PKA) or protein kinase C (PKC) decreases their activation threshold, leading to an increased open probability of the ion channels [80,81]. Besides this short-term regulation, intermediate and long-term regulatory mechanisms also lead to the sensitization of TRP receptors. Phosphorylation of TRPV1 protein stored in vesicles may accelerate their exocytosis and the insertion of functional TRPV1 receptors into the cell membrane (see below) [82]. Increasing the amount of TRPV1 or TRPA1 receptors on the surface membrane contributes to nociceptor sensitization on an intermediate time scale. Effects increasing receptor protein expression may have long-lasting effect on nociceptor sensitivity [83,84].

Activation of TRPV1 and TRPA1 receptor channels by pungent plant compounds like capsaicin or substances from mustard and garlic as well as environmental irritants such as formaldehyde and acrolein [85] go along with inward sodium currents depolarizing the sensory endings and calcium currents inducing neuropeptide release (Figure 2) [38], which leads to arterial vasodilatation and increased blood flow [86,87,88]. One might assume that the released neuropeptides contribute to nociceptor sensitization. However, CGRP is not activating nociceptors directly and does therefore not cause pain but seems to contribute only to pain generation after the nociceptive system has already been sensitized, for example by glycerol trinitrate (GTN), which mimics the action of nitric oxide (NO), as it was demonstrated by CGRP injection into facial skin in GTN pretreated rats [89].

Neurogenic inflammation of the meningeal tissue and CSD are pathophysiological conditions considered as endogenous mechanisms initiating cascades of events that may activate and/or sensitize nociceptors in the meningeal tissue leading to hypersensitivity and allodynia. In animal models chemical irritation of the dura mater was found to activate and sensitize meningeal nociceptors [59,90]. Proinflammatory substances play a significant role in these sensitizing processes (Figure 2). Initial release of neuropeptides from activated nociceptors may potentiate the secretion of proinflammatory mediators, e.g., by degranulating dural mast cells [91,92]. Electrophysiological recordings showed that capsaicin-induced neuronal responses are enhanced by prostaglandine E2 and prostacyclin through the activation of cAMP-dependent protein kinase A (PKA) and protein kinase C (PKC) pathways [80,93]. PKC activation and consequent phosphorylation seem to be also mechanisms of ATP- and bradykinin-induced sensitization of nociceptors [94,95].

Chronic migraine patients show elevated levels of NGF in their cerebrospinal fluid [96]. Under experimental conditions expression of NGF mRNA and release of NGF can be enhanced by sensory neuropeptides like CGRP [97]. Elevated NGF levels, similarly to insulin and insulin-like growth factor 1 (IGF-1), activate phosphatidylinositol-3-kinase that may lead to phosphorylation of the TRP receptors through calcium/calmodulin-dependent protein kinase II (CaMKII) and PKC [98,99].

Activation of the tyrosine kinase receptor A (TrkA) by NGF, insulin or IGF-1 initiates a cascade of events leading to phosphorylation of TRPV1 that is located in intracellular vesicles. This increases the amount of functional TRPV1 in the cell membrane by increasing its trafficking [100]. Recent experiments showed upregulation of TRPA1 mRNA in trigeminal ganglion neurons innervating the dura mater after chronic exposure of rats to an atmosphere containing the TRPA1 agonist acrolein [101]. Interestingly, an increase in TRPV1 protein content but not TRPV1 mRNA was measured following treatment of trigeminal ganglion neurons with NGF mediated by the mitogen-activated protein kinase pathway [102].

Calcium inflow into the nociceptors may also lead to desensitization of TRPV1 via calcium/calmodulin-dependent protein phosphatase 2B (calcineurin) and β-arrestin-2 [103,104]. Similar mechanisms may be assumed for TRPA1. Thus, sensitization and desensitization counteract and may be more or less balanced depending on the state of TRP activation (Figure 2). The situation is even more complicated in neurons expressing both TRPV1 and TRPA1 [52]. There is evidence that these two receptor channels interact in that TRPA1 activation limits TRPV1-mediated current but also desensitization due to the TRPA1-mediated current [105]. Therefore, it seems possible that the interactions of both channels can both increase or decrease trigeminal nociceptor activity, depending on the strength of their activation. Extracellular recordings from meningeal afferents revealed a mainly cooperative effect between TRPV1 and TRPA1 confirmed in TRPV1-deleted mice [55]. Activation of TRPA1 receptor channels increased the activation threshold and did not cause propagated action potentials unless TRPV1 was present. These effects may be further modulated in afferent second order neurons. In a rat model of meningeal nociception, recordings from neurons in the spinal trigeminal nucleus with meningeal afferent input indicated that individual neurons may be activated or inhibited by the TRPA1 agonist nitroxyl (HNO, see below) [56]. Thus, the effects of TRPA1 agonists may depend on the site of action (peripheral vs. central terminals) and the functional characteristics of higher order neurons.

Single nucleotide polymorphisms associated with the susceptibility for migraine have been identified in genes coding for TRPV1 and TRPV3 but the pathophysiological relations with known migraine mechanisms are yet unclear [106]. Besides TRPV1 and TRPA1, TRPM8 receptors are expressed in TG neurons (Figure 2). TRPM8 is interesting, because genome-wide association studies showed that it may be implicated in migraine [107,108], i.e., more specifically, single-nucleotide variants near or within the TRPM8 gene resulting in reduced TRPM8 expression are associated with reduced risk for migraine [109]. TRPM8 has been identified as a sensor for environmental cold [57] and low body temperature [110] but experimental work on trigeminal nociceptive functions is contradictory. The TRPM8 agonist icilin applied onto the rat cranial dura mater induced cutaneous facial and hind paw allodynia that was attenuated by systemic pretreatment with a TRPM8 antagonist [111,112]. On the contrary, TRPM8 activation reversed an increase in facial sensitivity to heat induced by meningeal inflammation, and in a trigeminal ganglion cell assay TRPM8 activation inhibited TRPV1 effects [101]. Thus TRPM8 activation by exogenous agonists may both aggravate and alleviate headache-related behaviors, possibly depending on the activation of other pro-nociceptive receptors of meningeal afferents [58].

In this context it is very interesting that both TRPV1 and TRPM8 receptor channels are differently involved in the regulation of body temperature [109]. While TRPV1 activation causes a decrease, TRPM8 activation causes an increase in body temperature; the basal activity of both receptor channels counteracts excesses of the body temperature, thereby contributing to homeostasis. It is intriguing, although evolutionary fitting, that single-nucleotide variants causing reduced TRPM8 expression, which is associated with a higher risk for migraine, are more frequently found in populations that live in warmer geographic areas [113].

Ten years ago already, several TRPV1 antagonists have entered clinical trials, including ABT-102, SB-705498, AMG-517, MK2295 and GRC-6211 [114], however, the major setback in the development of TRPV1 antagonists for the treatment of chronic pain was an elevation of the body temperature. Attempts to develop TRPV1 antagonists without causing hyperthermia are in the focus of recent pharmacological research [115].

## 7. Sensitization of Trigeminal Nociceptors Following Cortical Spreading Depression

Cortical spreading depression (CSD) is characterized by a slowly propagating wave of depolarization followed by suppression of cortical activity, a complex event, which induces dramatic changes in neural and vascular functions. CSD has been implicated in the pathophysiology of migraine, derived from neuroimaging studies which showed changes in cortical blood flow (propagating with similar velocity as the experimental CSD) during the visual aura phase of migraine [116,117]. Since aura in most cases precedes the onset of headache, it was hypothesized that CSD may lead to the activation of meningeal nociceptors [118]. Although animal models provide support for this hypothesis, its relationship to migraine attacks is still under debate. CSD may stimulate dural nociceptors either by axon collaterals innervating both the pia mater and the dura mater [119] or by the direct diffusion of molecules such as potassium ions, hydrogen ions or glutamate released during CSD [120,121]. CSD may also increase the production of reactive oxygen species in the cortex, in meningeal tissues and also in the trigeminal ganglion [122]. Low extracellular pH or oxidizing agents may directly activate TRPA1 and TRPV1 channels through modification of cysteine (Figure 2); they excite trigeminal nociceptors and induce the release of CGRP from trigeminal terminals to promote sensitization of trigeminal neurons [123]. Recently it was shown that CGRP-binding monoclonal antibodies can prevent the development of central sensitization and cutaneous allodynia elicited by CSD [124].

In vivo two-photon imaging of anesthetized mice revealed changes in the morphology of macrophages and motility of dendritic cells in the meninges in relation to CSD [125]. During CSD, meningeal macrophages changed their shape and migration of dendritic cells stopped, which was considered as changes in meningeal immune cell function signalizing to the TRPV1 expressing meningeal nociceptors. These observations are consistent with earlier findings on meningeal immune cells, where CSD was observed causing degranulation of mast cells and potentiating the activation of dural nociceptors [126].

## 8. Role of Metabolic States in Trigeminal Sensitization

Population-based studies indicate that migraine can be associated with metabolic disorders. Obesity, insulin resistance and diabetes mellitus are conditions that are linked to the primary headache migraine. These conditions may enhance the frequency and severity of headache attacks and they may increase the risk for migraine [127,128]. Our knowledge about sensitization of trigeminal nociceptors in metabolic disorders is based on studies in experimental animals.

Recent experimental observations indicated that in rats, high-fat high-sucrose (HFHS) diet increased fasting blood glucose and insulin concentrations as well as levels of the circulating proinflammatory cytokines interleukin-1β (IL-1β) and interleukin-6 (IL-6). HFHS diet-induced obesity was associated with enhanced basal and stimulated CGRP release from meningeal nociceptors. Both TRPV1 and TRPA1 receptor sensitivity to the specific stimulatory agents capsaicin and acrolein, respectively, was increased. Stimulation of TRPV1 and TRPA1 receptors resulted also in a significantly augmented vasodilatory response in meningeal blood vessels of obese animals [129,130].

Experimental results indicate that migraine pathophysiology and the conditions leading to the excess of body fat overlap in several aspects. Adipose tissue produces bioactive molecules, which serve as regulators of metabolism and also modulate immune functions [131,132]. Since HFHS diet of experimental animals sensitizes TRP receptor function without obvious changes in density or distribution of TRPV1-immunoreactive afferents of the dura mater and TRPA1 protein expression in the trigeminal ganglion, sensitization of the TRP receptors is more likely the consequence of the release of proinflammatory agents produced by the adipose tissue. IL-1β may directly modify the gating properties of TRP channels for noxious stimuli in primary sensory neurons but leave the transport of TRP channels to the plasma membrane unaffected [133].

Streptozotocin injection destroying insulin secreting cells of pancreatic Langerhans islets reduced the number of TRPV1-immunoreactive nerve fibers of the dura mater in rats [134]. Impairment of trigeminal nociceptors develops slowly; loss of TRPV1-mediated CGRP release and the lack of meningeal vasodilatation upon TRPV1 stimulation cannot be observed shortly after the induction of diabetes, which may be explained by the slow decrease in neuronal peptide levels after treatment with streptozotocin. In the rat trigeminal ganglion, CGRP markedly declined only five weeks after the induction of diabetes [135].

Despite the impaired function of chemosensitive trigeminal afferents, clinical observations indicate that diabetic patients suffer more frequently from headaches than non-diabetics [127]. It was hypothesized that dysfunction of the afferents may contribute to the enhanced incidence of headaches in diabetics due to a limited elimination of tissue metabolites and inflammatory mediators from meningeal tissues.

Another endocrine disorder, hypothyroidism, seems to have a bidirectional association with migraine. Common genetic and immune mechanisms are considered as underlying pathophysiological processes [136]. TRP receptor ion channels may function as possible targets for proinflammatory cytokine effects leading to increased incidence of migraine attacks in hypothyroid patients.

## 9. Intraganglionic Mechanisms Involved in Nociceptor Sensitization

Migraine attacks can be triggered by the activation of trigeminal afferents innervating extracranial tissues. Inhaled environmental irritants stimulating TRPA1 receptors of the nasal mucosa, allergic rhinitis, acute sinusitis and temporomandibular joint disorder are risk factors for migraine [101,137]. Cross-excitation within the trigeminal ganglion may explain why activation of one branch of the trigeminal nerve can promote cellular changes that result in sensitization within the entire ganglion and lead to the generation of headaches.

CGRP can be released not only from the peripheral and central terminals of activated trigeminal neurons but also from the cell body [138]. CGRP released within the ganglion may facilitate spreading of information throughout the whole ganglion [139]. Within the trigeminal ganglion both neurons and satellite glial cells can express CGRP receptor components, and accordingly they serve as targets of CGRP effects. In rats 32% of neurons in the trigeminal ganglion express both calcitonin-like receptor (CLR) and receptor activity modifying protein 1 (RAMP1) receptor components indicating the formation of functional CGRP receptors [65]. Neurons expressing CGRP receptor components are frequently found in close vicinity to CGRP containing neurons, which enable cross-talk between them. The neurotransmitters glutamate and ATP have also been implicated in the communication within the trigeminal ganglion. Experimental studies have demonstrated the expression of excitatory glutamate receptors and ATP receptors in sensory neurons [140,141].

Besides direct transmission of information from one neuron to the other, activation of satellite glial cells may provide an additional indirect mechanism for the sensitization of other neurons initially not involved in the nociceptive process. Glial cells activated by the initial transmitter release from trigeminal neurons may release inflammatory molecules leading to sensitization of neurons [142]. Glutamate release from satellite glial cells cultured from the trigeminal ganglion was also reported as intraganglionic signaling mechanism [143].

The TRPV1 receptor agonist endovanilloid/endocannabinoid anadamide and other endogenous lipid metabolites or inhaled irritants activating trigeminal TRPA1 receptors are well-known mechanisms leading to activation of some trigeminal neurons [54,144]; their intraganglionic effect may probably play a significant role in the process of cross-sensitization within the trigeminal ganglion.

## 10. Sensitization Processes within the Trigeminovascular System

A subpopulation of trigeminal neurons innervating the dura mater express the proteinase-activated receptor-2 (PAR-2) that may be a target for proteolytic enzymes, e.g., tryptase released from activated mast cells [145]. Cleavage exposes tethered ligand domains that bind to and activate cleaved receptors. Since meningeal mast cells are targets for neuropeptide actions, release of CGRP or substance P from meningeal afferents may degranulate mast cells leading to the release of mast cell tryptase and consequent PAR-2 activation. Experimental observations indicate that activation of PAR-2 induces sensitization of the TRPV1 receptor in trigeminal afferents, elicits CGRP release and enhances meningeal blood flow upon stimulation the TRPV1 receptor with its specific agonist capsaicin [146]. Mast cell tryptase activating the PAR-2 receptor may sensitize not only TRPV1 but also TRPA1 receptors of trigeminal afferents. Cleavage of PAR-2 may activate phopholipase C, which unlocks TRP receptors from phosphatidylinositol-4,5-bisphosphate inhibition [147]. Since also mast cells possess PAR-2 receptors, a mutual activation of mast cells through the released tryptase may initiate a self-triggering mechanism of mast cells resulting in an amplification of the primary nociceptive response and probably also the sensitivity of the second-order neurons in the nociceptive pathway [148].

Hydrogen sulfide (H_2_S) and NO, members of the gasotransmitter family, are involved in the regulation of a great variety of physiological functions, including cardiovascular functions, nociception and inflammation [149,150]. A substantial body of evidence indicates that H_2_S increases the firing rate of trigeminal ganglion neurons by acting on TRP receptors [56,151]. H_2_S is synthesized from cysteine through several enzymatic pathways. The H_2_S producing enzyme cystathionine β-synthase (CBS) is abundantly expressed in the trigeminal ganglion of rats [152] and under inflammatory conditions CBS expression may be upregulated at both protein and mRNA levels [153].

Recent studies reported that H_2_S produced in peripheral tissue may interact with NO and results in generation of polysulfides [154,155] or HNO, a protonated, one electron-reduced derivative of NO [156]. Polysulfides and HNO are activators of TRPA1 receptors. In the dura mater HNO production and its effect on TRPA1 receptors seems to be the main mechanism of H_2_S-induced vasodilatation and CGRP release [157]. In the trigeminal ganglion of rats, CBS immunoreactivity was partly colocalized with TRPA1 in small trigeminal neurons [152]. Continuous generation of HNO in the meningeal tissue and its CGRP releasing effect through the activation of TRPA1 seems to be also a significant vasodilatory component maintaining basal meningeal blood flow [157].

## 11. Medication-Induced Trigeminal Nociception and Pain

Epidemiological studies indicate that in patients suffering from primary headaches the progressive increase in the use of acute medication increases the number of headache days per months leading to medication overuse headache (MOH). All specific migraine and pain medications seem to have the capacity to cause MOH [158]. Pathophysiological studies in animal models have suggested that central sensitization of the trigeminal nociceptive pathway may function as a basis for MOH. Central sensitization induced by chronic application of drugs like triptans, paracetamol or opiates seems to underlie the pathogenesis of MOH.

A recent study reports the lack of association between the polymorphisms of TRPV1, TRPA1 and TRPM8 ion channels and MOH in patients [159]. In different animal models of MOH, similar metabolic changes in the trigeminovascular system were observed; increase in CGRP expression and decrease in 5-HT1B/D receptor expression [160]. Clinical studies in MOH patients have shown that 5-HT levels decrease, which may subsequently reduce the inhibitory effect mediated by the 5-HT1B/1D receptors of trigeminal sensory neurons on sensory neuropeptide release [161]. Increased CGRP release upon TRPV1 or TRPA1 activation induced by their specific agonists may be involved in the sensitization process.

Drugs used for other purposes than headache therapy may alter the sensitivity of nociceptors leading to uncomfortable or painful sensations upon administration. The α1-adrenoceptor agonist phenylephrine used at high concentrations as a mydriatic agent and for the treatment of nasal congestion may induce local burning sensation or headache as adverse side effect [36]. Recent experiments revealed that high concentrations of phenylephrine activate trigeminal neurons in rodent models of trigeminal nociception. This activation induces calcium inflow and consequent CGRP release from trigeminal neurons innervating the meningeal tissues. As a neurovascular consequence of the peptide release, increases in meningeal blood flow were recorded. Activation of trigeminal nociceptors was mainly the result of the activation of TRPV1 receptor channels by phenylephrine. The contribution of TRPA1 receptor activation was excluded, since pretreatment of the dura mater with the TRPV1 receptor antagonist abolished the phenylephrine-induced CGRP release and meningeal blood flow increase, while the TRPA1 receptor antagonist had no effect on it. Results obtained in trigeminal neurons of TRPV1-deficient animals supported this observation. Phenylephrine-induced calcium transients and CGRP release were abolished in trigeminal ganglion neurons of TRPV1-deficient animals [36].

A potent anthracycline-type antitumor agent, adriamycin, is used in the treatment of various malignancies [162]. As serious side effects, impairments in sensory nerve functions were reported, which may also significantly contribute to the cardiomyopathy developing in some patients [163,164]. Recent findings revealed multiple but selective impairments of receptor functions in the trigeminovascular system after adriamycin treatment. Adriamycin affected the nociceptor channels TRPV1 and TRPA1 and also the CGRP receptors of meningeal arteries. While the impairment of chemosensitive nociceptors was clearly indicated by the reduced TRPV1 content of trigeminal ganglia of adriamycin-treated animals, in the dura mater whole mount preparations the density and distribution of TRPV1-immunoreactive afferents were not significantly changed. Measurements of CGRP release in an ex vivo dura mater preparation revealed an altered dynamic of peptide release upon repeated stimulations of TRPV1 and TRPA1 receptors. The first application of an agonist of TRPV1 or TRPA1 receptor induced a significantly higher increase in CGRP release in adriamycin-treated animals compared to control animals, while further applications failed to increase the release [165]. Although headache is not a major problem among patients treated with adriamycin, and to our knowledge no clinical studies indicated increased incidence of headache in this population, the impaired trigeminal nociceptor function coupled with impaired vasodilator capacity of meningeal blood vessels may be an important mechanism contributing to pain sensation.

## 12. Synopsis: Relevance of TRPV1 and TRPA1 in Primary Headaches

Clinical observations and animal studies provide evidence for a central role of trigeminal afferents in the mechanisms of primary headaches [5,166]. A significant population of trigeminal afferents expressing the nociceptive ion channels TRPV1 and TRPA1 are peptidergic. They contribute not only to the transmission of the nociceptive information towards the central nervous system but their neuropeptide content released from their peripheral and central terminals and also from their cell body upon activation may have a significant modulatory effect on long-term pain perception. Different peptides (CGRP, substance P or pituitary adenylate cyclase-activating peptide) possessing vasodilatory function have been identified in trigeminal neurons [167,168]. Being the most abundant peptide in the trigeminal system, expressed by approximately 40% of neurons, CGRP has gained major attention in clinical and experimental studies on the pathomechanisms of primary headaches. Reducing the release of CGRP, blocking its receptor or binding CGRP with humanized anti-CGRP antibodies seem to be effective in the treatment or prevention of migraine attacks [76,169]. CGRP released within the trigeminal ganglion may sensitize other neurons directly or by acting on satellite glial cells. According to the currently accepted view, meningeal arterial vasodilatation induced by CGRP does not play a causative role in primary headaches [5]. CGRP-induced meningeal vasodilatation may still have a beneficial effect, e.g., by removing inflammatory mediators from the meningeal tissue and preventing or reducing the activation and/or sensitization of the nociceptive pathway. Release of CGRP from trigeminal nociceptors and initiation of processes leading to peripheral and central sensitization of the nociceptive pathway can be induced by different endogenous and exogenous agents acting on TRPV1 or TRPA1 receptors. The important role of TRP receptors in the activation and sensitization of trigeminal nociceptors make them possible targets for drug developments that counteract the channel opening or the expression of the channels, thereby reducing CGRP release from trigeminal afferents. Removing the key mediator CGRP may interrupt the vicious circle leading to increased susceptibility for headache attacks.

Given that TRPV1 receptor channels play a major role in nociceptive transduction, the question was if they are also involved in the generation of primary headaches. Therefore, animal models of headache have been employed, which admittedly provided inconsistent results. Systemic administration of the TRPV1 antagonist SB-705498 suppressed responses to stimulation of the dura mater and the facial skin and reversed the sensitization of second order neurons in the spinal trigeminal nucleus of cats induced by inflammatory compounds [170]. The authors concluded that inhibiting TRPV1 may be useful to treat inflammatory trigeminovascular pain but expressed their skepticism regarding primary headaches. In two other animal studies in the rat the TRPV1 antagonists JNJ-38893777 and JNJ-17203212 suppressed the generation of the immediate early gene c-fos, provoked by intracisternal application of inflammatory mediators, and attenuated the CGRP release into the external jugular vein following injection of capsaicin into the carotid artery. The authors concluded that TRPV1 may play a role in the pathophysiological mechanisms relevant to migraine [171]. In contrast, the TRPV1 antagonist A-993610 had no significant effect on the activity of rat spinal trigeminal neurons evoked by electrical stimulation of the dura mater, did not block neurogenic vasodilatation of dural arteries and did not cause any change in the CSD response induced by pin prick. Based on these results the authors concluded that blockade of TRPV1 receptor channels will gain no role in the treatment of acute migraine [172].

Some volatile agonists of TRPA1 are known to trigger headaches in sensitive persons. The best known example is the scent of the Californian “headache tree”, Umbellularia california, the active compound of which, the monoterpene ketone umbellulone, was found to activate TRPA1 receptor channels expressed in HEK293 cells and trigeminal ganglion neurons and to cause nociceptive behavior in wild-type but not TRPA1-deficient mice [54,173]. Another plant is fewerfew (Tanacetum parthenium), which has been used for centuries to treat headache and other pains. Its major constituent, parthenolide, has also been found to stimulate TRPA1 receptor channels but behaves as a partial agonist, which preferably desensitizes trigeminal neurons [174]. Curcumin, the active principle of turmeric root (Curcuma longa) also activates and subsequently desensitizes TRPA1 ion channels [175]. Regarding TRPV1, partial agonism was found for two major components of Evodia rutaecarpa, evodiamine and rutaecarpine, from which the first one reduced capsaicin- and proton-evoked currents in TRPV1-expressing HEK293 cells [176].

Extracts of the butterbur plant (Petasites hybridus) with the main component isopetasin have long been used as preventives for migraine [177]. Isopetasin has been shown to induce calcium signals and inward currents in rodent trigeminal ganglion neurons and CGRP release from mouse dorsal spinal cord via activation of TRPA1 receptor channels, while pre-exposure to isopetasin attenuated responses to the TRPA1 agonist allyl isothiocyanate and the TRPV1 agonist capsaicin in these preparations. Repeated systemic administration of isopetasin attenuated also mouse facial rubbing evoked by local allyl isothiocyanate or capsaicin. The authors concluded that activation of TRPA1 channels by isopetasin and excitation of neuropeptidergic trigeminal neurons causes heterologous neuronal desensitization, which may account for the anti-migraine effect of petasites extracts [178]. Thus, regarding the above discussion, the role of TRP receptor channels in headache generation seems to be ambiguous, and it may be a matter of the balance of sensitizing-desensitizing mechanisms if a TRP agonist is preferentially triggering or preventing headaches.
